# A Real-Time Stability Control Method Through sEMG Interface for Lower Extremity Rehabilitation Exoskeletons

**DOI:** 10.3389/fnins.2021.645374

**Published:** 2021-04-13

**Authors:** Can Wang, Ziming Guo, Shengcai Duan, Bailin He, Ye Yuan, Xinyu Wu

**Affiliations:** ^1^Guangdong Provincial Key Lab of Robotics and Intelligent System, Shenzhen Institute of Advanced Technology, Chinese Academy of Sciences, Shenzhen, China; ^2^Shenzhen Institute of Advanced Technology, University of Chinese Academy of Sciences, Shenzhen, China; ^3^CAS Key Laboratory of Human-Machine Intelligence-Synergy Systems, Shenzhen Institute of Advanced Technology, Shenzhen, China; ^4^Guangdong-Hong Kong-Macao Joint Laboratory of Human-Machine Intelligence-Synergy Systems, Shenzhen Institute of Advanced Technology, Chinese Academy of Sciences, Shenzhen, China; ^5^Department of Mechanical and Automation Engineering, The Chinese University of Hong Kong, Hong Kong, Hong Kong

**Keywords:** rehabilitation exoskeleton robot, real-time motion stability, gait switch, surface electromyography, motion intention recognition, muscle fatigue, ergonomic effects

## Abstract

Herein, we propose a real-time stable control gait switching method for the exoskeleton rehabilitation robot. Exoskeleton rehabilitation robots have been extensively developed during the past decade and are able to offer valuable motor ability to paraplegics. However, achieving stable states of the human-exoskeleton system while conserving wearer strength remains challenging. The constant switching of gaits during walking may affect the center of gravity, resulting in imbalance of human–exoskeleton system. In this study, it was determined that forming an equilateral triangle with two crutch-supporting points and a supporting leg has a positive impact on walking stability and ergonomic interaction. First, the gaits planning and stability analysis based on human kinematics model and zero moment point method for the lower limb exoskeleton are demonstrated. Second, a neural interface based on surface electromyography (sEMG), which realizes the intention recognition and muscle fatigue estimation, is constructed. Third, the stability of human–exoskeleton system and ergonomic effects are tested through different gaits with planned and unplanned gait switching strategy on the SIAT lower limb rehabilitation exoskeleton. The intention recognition based on long short-term memory (LSTM) model can achieve an accuracy of nearly 99%. The experimental results verified the feasibility and efficiency of the proposed gait switching method for enhancing stability and ergonomic effects of lower limb rehabilitation exoskeleton.

## 1. Introduction

In recent years, powered lower limb exoskeleton robots have been proven to be particularly versatile and effective in the medical and military fields. Lower-limb exoskeleton robots can be roughly divided into two categories, including the auxiliary exoskeleton robot and the assisted exoskeleton robot. The auxiliary exoskeleton robot is used to aid the sick and the elderly in their normal daily life functioning or medical rehabilitation. The assisted exoskeleton robot is utilized for strength augmentation in disaster rescues or auxiliary operations. The auxiliary exoskeleton is our discussed object in this investigation, although the mechanism design and manufacture, path planning, and high-level intent control for lower limb exoskeleton robot has been developed to a relatively sophisticated level. Unfortunately, achieving a stable gait switching during walking for lower limb exoskeleton robots is still a challenging research.

Research into exoskeleton robots has resulted in considerable development of lower limb rehabilitation exoskeleton robots. Auxiliary walking exoskeleton robots are mainly applied in the field of medical rehabilitation for patients with inferior legs and feet, such as the elderly and disabled. Some heterogeneous structure exoskeleton robots (Li et al., 2015; Parietti et al., 2015; Eguchi et al., 2018) are investigated in recent years. However, it was found that those exoskeleton robots take advantage of the heterogeneous structure with human lower limbs to gain walking stability, which may have some support strength but poor adaptability. As opposite of heterogeneous exoskeleton robots, most paraplegic rehabilitation exoskeleton robots have developed to be humanoid bipedal robots, and some of these exoskeletons are equipped with crutches to maintain better balance. Exoskeleton robots require the human interface to provide walking stability so that it may function as a flexible assistant. Such robots have been developed by commercial companies including Rewalk (Talaty et al., 2013), Ekso Bionics (Baunsgaard et al., 2018), Ailegs (Chen et al., 2018), and MaiBu BEAR H1. Exoskeleton robots especially require the walking stability as far as possible so as to provide flexible assistant. The Hybrid Assistive Limb (HAL) (Shimizu et al., 2019), which takes advantage of sEMG in control strategy, was designed by the robotics company Cyberdyne (Yilmaz et al., 2018).

Moreover, plenty of research institutes and universities are also actively developing humanoid exoskeletons. Sogang University in Korea developed a exoskeleton namely SUBAR (Chen et al., 2013; Hwang and Jeon, 2018), which could estimate the muscular torque of its wearer. Singapore's Nanyang Technological University (Mertz, 2012) and Harvard University (Abe et al., 2018) have also made solid progress in the development of assisted exoskeletons. The Chinese University of Science and Technology designed an exoskeleton robot driven by a servo motor (Li et al., 2019) and developed a fuzzy algorithm for lower extremity exoskeleton (Huang et al., 2016a). The exoskeleton team of H. C. from University of Electronic Science and Technology of China have developed exoskeletons for paraplegia (Huang et al., 2018), hemiplegia patients (Peng et al., 2020), and human-power augmentation (Huang et al., 2016b, 2019). The exoskeleton group at the Shenzhen Institutes of Advanced Technology of the Chinese Academy of Sciences has developed the fourth generation of the SIAT exoskeleton robot. With four degrees of freedom (DoFs), this exoskeleton robot has successfully enabled persons with disabilities to stand up and walk independently (Liu et al., 2017; Wang et al., 2018a).

The human–exoskeleton interaction is drawing more and more attention (Huang et al., 2016b,c; Lin et al., 2019). The neural interface between the human and exoskeleton based on the brain computer interface (BCI) (Yuan et al., 2018) and sEMG has been a hot research topic. Brain–computer interfaces are relatively unstable (Wang et al., 2018b). Decoding sEMG signal is one of the important approaches for intention identification (Chu et al., 2007). The human–exoskeleton system is operated by the identified results of decoding sEMG signals, which can prevent the disadvantages of the operation with physical buttons. For instance, the pilot can focus on maintaining balance rather than pressing control button to operate exoskeleton at the same time, which may result in fatigue and instability. In addition, the sEMG is an intuitive and noninvasive way to real-time monitor the muscle states, which has proven to be an effective in muscle fatigue detection (Lin et al., 2019).

**Figure 1:**
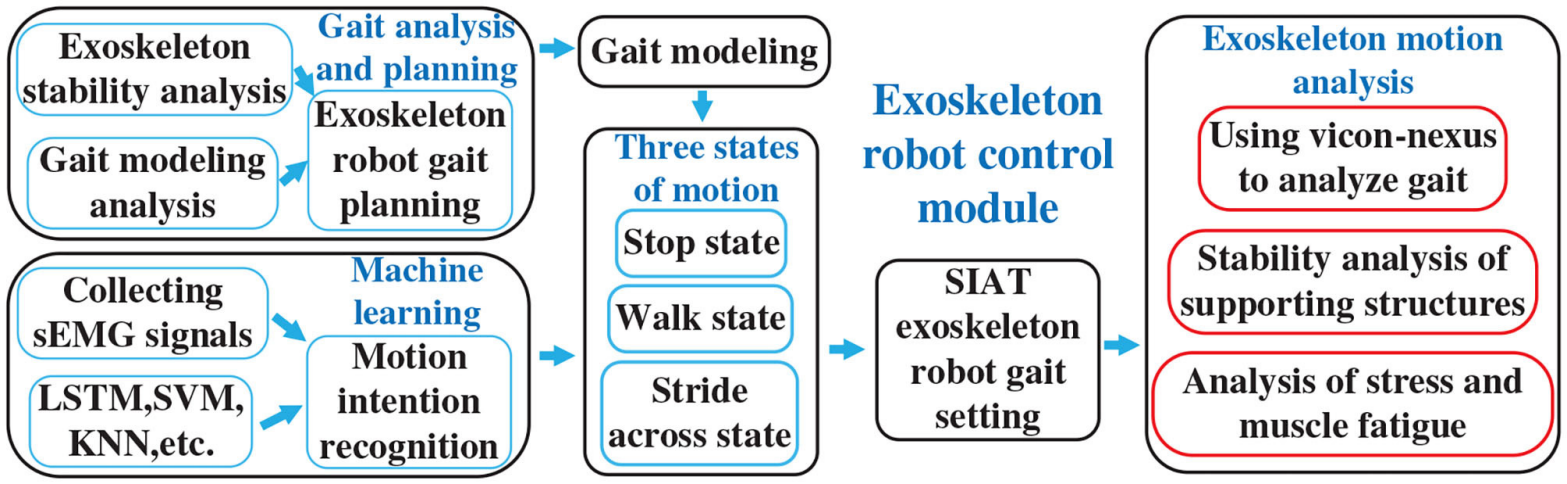
Rehabilitation exoskeleton motion analysis system.

While investigating the existing exoskeleton control methods, we found very few studies that have examined the minimum force requirements into the control scheme by the wearer. We also found scarce consideration for the state of the wearer while operating a lower limb exoskeleton. In detail, the use of crutches can lead to walking instability, thereby impacting exoskeleton assistance, while also impacting the patients' overall rehabilitation. In this study, the usage of crutches and gait switching is investigated to gain the stability of human-exoskeleton system. This study could be applied to rehabilitation training in rehabilitation medical institutions, which is customized for each patient. An overview of the developed rehabilitation exoskeleton motion analysis system is presented in Figure 1. As shown in Figure 1, the gait model based on stability analysis and gait planning is first completed. Then intention recognition based on sEMG with machine learning is demonstrated. After that, the exoskeleton is driven with the control model. Finally, the exoskeleton motion analysis is demonstrated.

In this study, real-time gait conversion is achieved through neural interface and proposed gait switching method based on the SIAT exoskeleton rehabilitation robot. The gait switching method trying to make two crutch-supporting points and a supporting leg form an equilateral triangle base to increase the stability of human-exoskeleton system and enhance its ergonomic effects, including joints load bearing and less muscle fatigue. The main contributions of this paper are as follows:

(1) The analysis of gait planning is demonstrated based on a human kinematics model and real-time motion stability based on the zero-moment point (ZMP).
(2) A neural interface is constructed based on sEMG to achieve motion intention recognition and muscle fatigue estimation.
(3) The stability of human–exoskeleton system and ergonomic effects of the proposed gait switching method are verified by the organized experiments.

The remainder of this paper is organized as follows. Section 2 provides methodology used in this research. Section 3 presents system setup. Section 4 details experimental works and results. Section 5 shows the conclusions and future work.

## 2. Methodology

### 2.1. Gait Analysis

#### 2.1.1. Gait Planning

**Figure 2:**
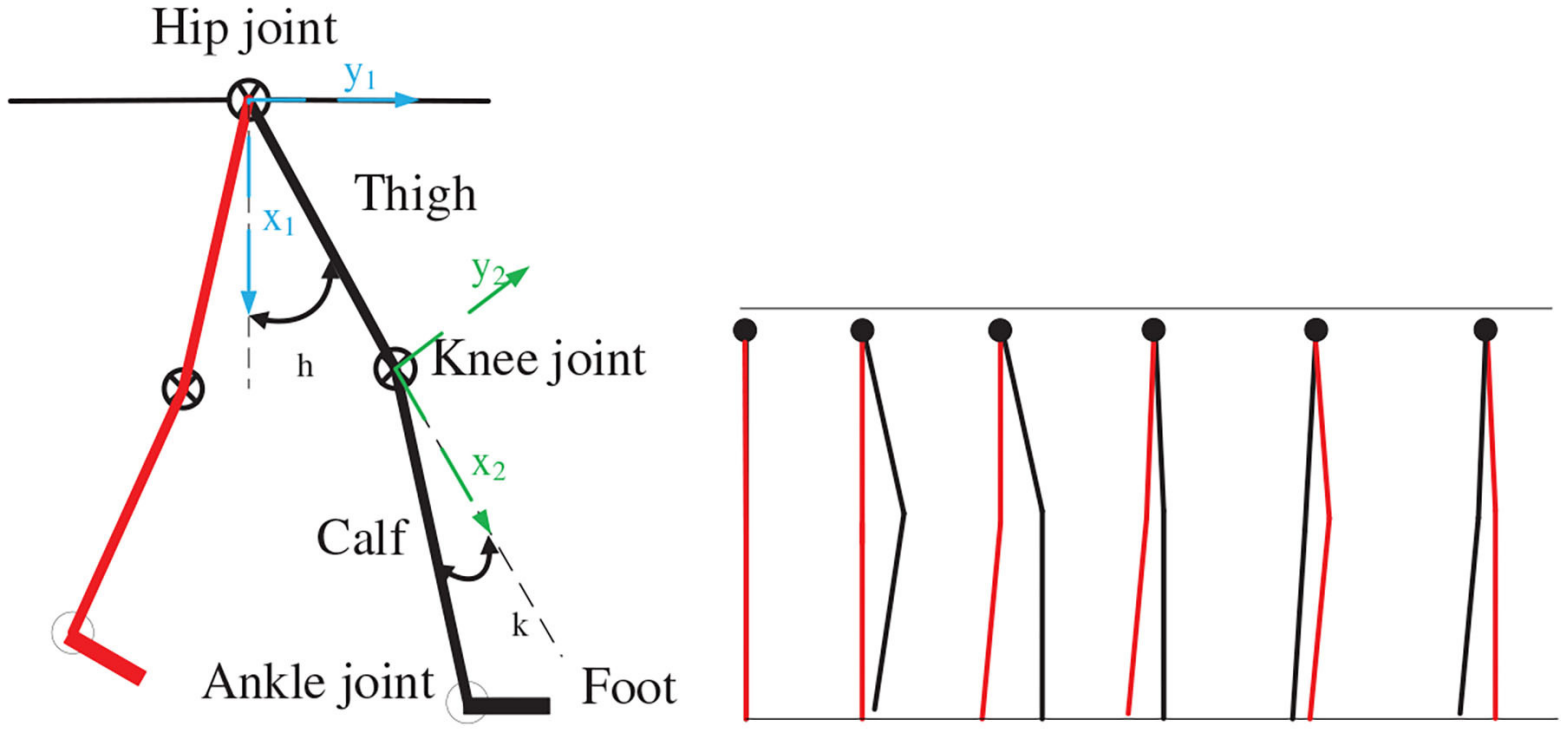
Lower extremity kinematics model.

In this study, the SIAT exoskeleton robot movement was achieved by the cooperation of the hip, knee, and ankle joints. We regard the lower limbs of the human body as a model of six connecting rods, as illustrated in Figure 2.

Taking the hip joint as the coordinate axis origin in the figure, the thigh, and calf are regarded as two straight rods in the sagittal plane. One end of the thigh is connected to the shank with the knee joint, and the foot is connected to the calf through the ankle joint. According to the angle relationship and adjacent coordinates, the Denavit–Hartenberg (DH) (Gillis et al., 2018) relationship between the hip, knee, and ankle joints can be determined, as indicated in Table 1. Through the DH relation, we can obtain the relational coordinate matrix as follows: where *cosθ*_1_ = *b*_1_, *cosθ*_2_ = *b*_2_, *sinθ*_1_ = *c*_1_, *sinθ*_2_ = *c*_2_, *cosα*_1_ = *j*_1_, *cosα*_2_ = *j*_2_, *sinα*_1_ = *k*_1_, *sinα*_2_ = *k*_2_.

(1) $$\begin{array}{r} A_{1}\;=\left[\begin{array}{cccc} b_{1} & -c_{1}j_{1} & c_{1}k_{1} & a_{1}b_{1}\\ c_{1} & b_{1}j_{1} & -b_{1}k1 & a_{1}c_{1}\\ 0 & k_{1} & j_{1} & d_{1}\\ 0 & 0 & 0 & 1 \end{array}\right] \end{array}$$

(2) $$\begin{array}{r} A_{2}\;=\left[\begin{array}{cccc} b_{2} & -c_{2}j_{2} & c_{2}k_{2} & a_{2}b_{2}\\ c_{2} & b_{2}j_{2} & -b_{2}k_{2} & a_{2}c_{2}\\ 0 & k_{2} & j_{2} & d_{2}\\ 0 & 0 & 0 & 1 \end{array}\right] \end{array}$$

(3) $$\begin{array}{r} T=A_{1}\times A_{2} \end{array}$$

(4) $$\begin{array}{r} T=\left[\begin{array}{cccc} b_{1}b_{2}-c_{1}c_{2}j_{1} & t_{01} & t_{02} & t_{03}\\ b_{2}c_{1}+b_{1}c_{2}j_{1} & t_{11} & t_{12} & t_{13}\\ c_{2}k_{1} & t_{21} & t_{22} & t_{23}\\ 0 & 0 & 0 & 1 \end{array}\right] \end{array}$$

where *t*_01_ = *c*_1_*k*_1_*k*_2_ − *b*_1_*c*_2_*j*_2_ − *b*_2_*c*_1_*j*_1_*j*_2_, *t*_02_ = *b*_1_*c*_2_*k*_2_ + *c*_1_*j*_2_*k*_1_ + *b*_2_*c*_1_*j*_1_*k*_2_, *t*_03_ = *a*_1_*b*_1_ + *a*_2_*b*_1_*b*_2_ + *c*_1_*d*_2_*k*_1_ − *a*_2_*c*_1_*c*_2_*j*_1_, *t*_11_ = *b*_1_*b*_2_*j*_1_*j*_2_ − *b*_1_*k*_1_*k*_2_ − *c*_1_*c*_2_*j*_2_, *t*_12_ = *c*_1_*c*_2_*k*_2_ − *b*_1_*j*_2_*k*_1_ − *b*_1_*b*_2_*j*_1_*k*_2_, *t*_13_ = *a*_1_*c*_1_ + *a*_2_*b*_2_*c*_1_ − *b*_1_*d*_2_*k*_1_ + *a*_2_*b*_1_*c*_2_*j*_1_, *t*_21_ = *j*_1_*k*_2_ + *b*_2_*j*_2_*k*_1_, *t*_22_ = *j*_1_*j*_2_ − *b*_2_*k*_1_*k*_2_, *t*_23_ = *d*_1_ + *d*_2_*j*_1_ + *a*_2_*c*_2_*k*_1_. The homogeneous coordinate matrix *T* that demonstrates the relationship between angle of hip and angle of ankle can be easily obtained through the homogeneous coordinate matrix *A*_1_ and *A*_2_. Based on the kinematics analysis of the exoskeleton robot, matrix relationships are used to calculate the positions of the hip and knee motors at each moment, which are converted into the motor rotation angle values.

**Table 1:** Denavit–Hartenberg relationship.

**Link**	**ai**	**αi**	**di**	**θi**
1	L1	0	0	θ_*h*_
2	L2	0	0	−θ_*k*_

**Figure 3:**
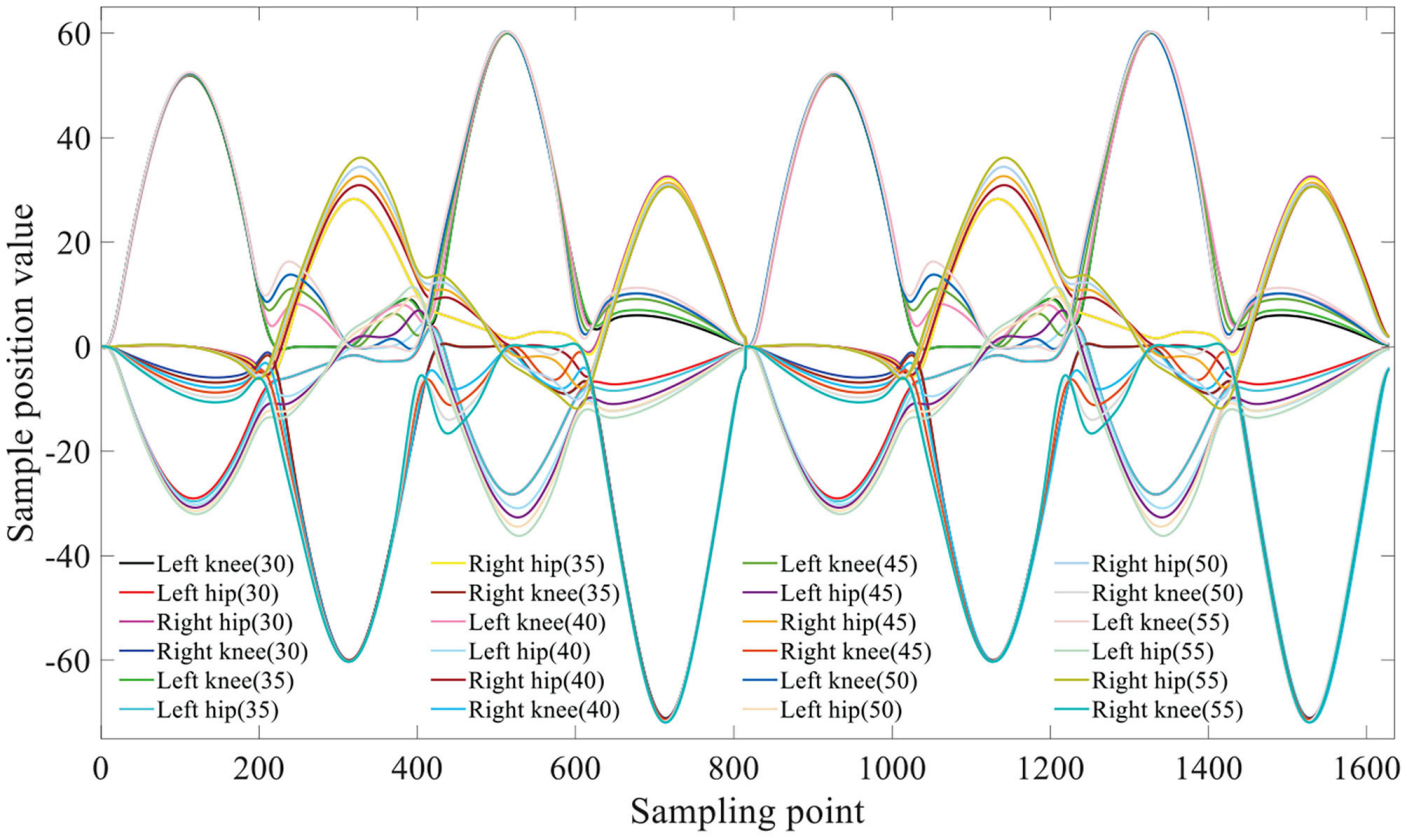
The motors gait trajectory.

The Vicon dynamic capture demonstrated that the joint exhibits a sinusoidal trajectory state when the human body moves. The SIAT rehabilitation exoskeleton has six DoFs: four actives and two passives. The hip and knee joints are initiative. We initially treat these two joints as two points and draw the trajectories of these points during the walking process to determine the trajectory approximating the sine function (Chen et al., 2019), as illustrated in Figure 3.

The sine function, *y* = *Asin*[(ω*x* + φ) + *k*], is used to express the joint movement trajectory of the human body. The hip joint is regarded as the coordinate 0 point, and the coordinate relationship is established according to the hip joint position. The length of the thigh is set to *l*_1_, the length of the calf is set to *l*_2_, and the number of sampling points is denoted by *i*. The sinusoidal relationship is used to obtain the hip joint abscissa *x*_0_(*i*) and ordinate *y*_0_(*i*). In the same manner, the abscissa *x*_3_(*i*) and ordinate *y*_3_(*i*) of the left foot position are obtained by means of the sinusoidal relationship (Guo et al., 2019).

The left knee position is determined as follows:

(5) $$\begin{array}{r} {\left(x-x_{3}\left(i\right)\right)}^{2}+{\left(y-y_{3}\left(i\right)\right)}^{2}=l_{2}^{2} \end{array}$$

(6) $$\begin{array}{r} {\left(x-x_{0}\left(i\right)\right)}^{2}+{\left(y-y_{0}\left(i\right)\right)}^{2}=l_{1}^{2} \end{array}$$

The maximum values of *x* and *y*, namely *x*_4_(*i*) and *y*_4_(*i*), respectively, are used. In this manner, the angle value of each position can be reversed as follows:

Left hip:

(7) $$\begin{array}{r} Ahl\left(i\right)=\text{arctan }\left(\frac{x_{4}\left(i\right)-x_{0}\left(i\right)}{y_{0}\left(i\right)-y_{4}\left(i\right)}\right) \end{array}$$

Left knee: takes 0 when the value is <0:

(8) $$\begin{array}{r} Akl\left(i\right)=\text{arctan }\left(\frac{x_{3}\left(i\right)-x_{4}\left(i\right)}{y_{4}\left(i\right)-y_{3}\left(i\right)}\right) \end{array}$$

Using the compilation algorithm, we determine the different positions of the motor at each moment, and these are combined into a complete motion cycle. The generated motor gait trajectory is illustrated in Figure 3. A parenthesis indicates the planning step size (in cm).

When compiling the control program, we set different step sizes, paces, and changed the motion states. The crutch support points were placed in different positions to form triangle support points of different shapes. In our previous experiments, we used the Tactilus^Ⓡ^ foot pressure insole to collect the pressure data and obtain the motion trajectory (Guo et al., 2019). It was verified that the stable triangle support point was more conducive to mastering balance. The present study uses the Vicon Nexus motion capture system in conjunction with the 3D force test runner to verify the results.

**Figure 4:**
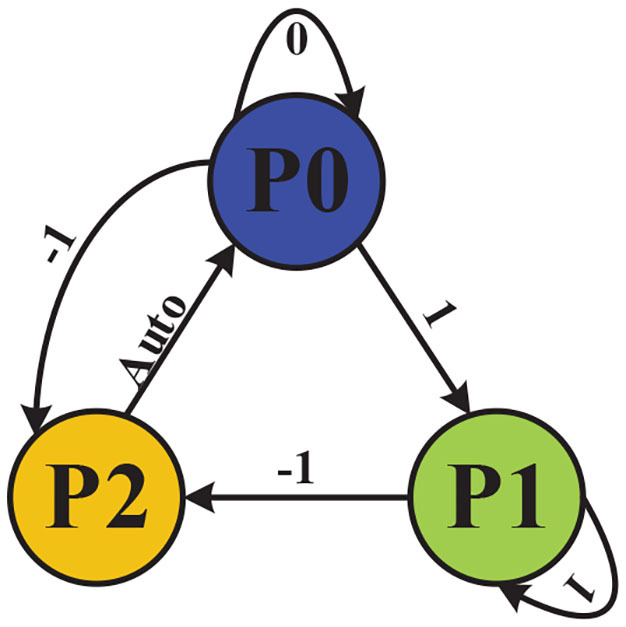
Control logic between exoskeleton states and intention recognition.

The control logic between exoskeleton states and intention recognition is illustrated in Figure 4. The three exoskeleton states are as follows: the quiescent state is set to P0 (stop), the walking state is set to P1 (walk), and step over an obstacle (21.5 * 13.5 * 10.5 cm) state is set to P2 (stride across). The starting state is P0 (stop).

In order to operate the exoskeleton robot, we set the control instruction value of detected immobility to 0, the control instruction value of detected walking to 1, and the control instruction value of detected crossing obstacle to −1, which is then reset to its initial state of P0 (stop). When the identified results of sEMG signals is 1, the exoskeleton would run to the state P1 (walk); when identified results of sEMG signals is 0, the exoskeleton would run to the state P0 (stop); when the identified results of sEMG signals is −1, the exoskeleton would run to the state P2 (stride across). The exoskeleton would automatically run to the state P0 (stop) after the P2 (stride across) state.

The motion intention is recognized through sEMG, which will be demonstrated in section 2.2.1. Because of the continuity and density of the collected sEMG, control instructions are sent to the exoskeleton only when three consecutive identical recognition results are detected. If three consecutive signals are not detected, the exoskeleton remains static after completing the current motion.

#### 2.1.2. Stability Analysis

To help paraplegic wearing the exoskeleton to master balance, a stable gait switch method based on the ZMP is proposed. For these patients, the lack of strength and sense of lower extremities means that they cannot rely on their legs to keep balance. Double crutches need to be added to ensure balance; hence, the exoskeleton exists in a quadruped state (Wu et al., 2018). By positioning the crutches to form a triangle base of support, additional stability is produced.

The walking process can be divided as follows: 40% of situations are supported by one leg, 20% of situations are supported by two legs, and the remaining 40% of situations are supported by one leg, the other is semi-supported. In order to evaluate the performance of the stable gait switch method, a real-time gait was divided into eight parts.

**Figure 5:**
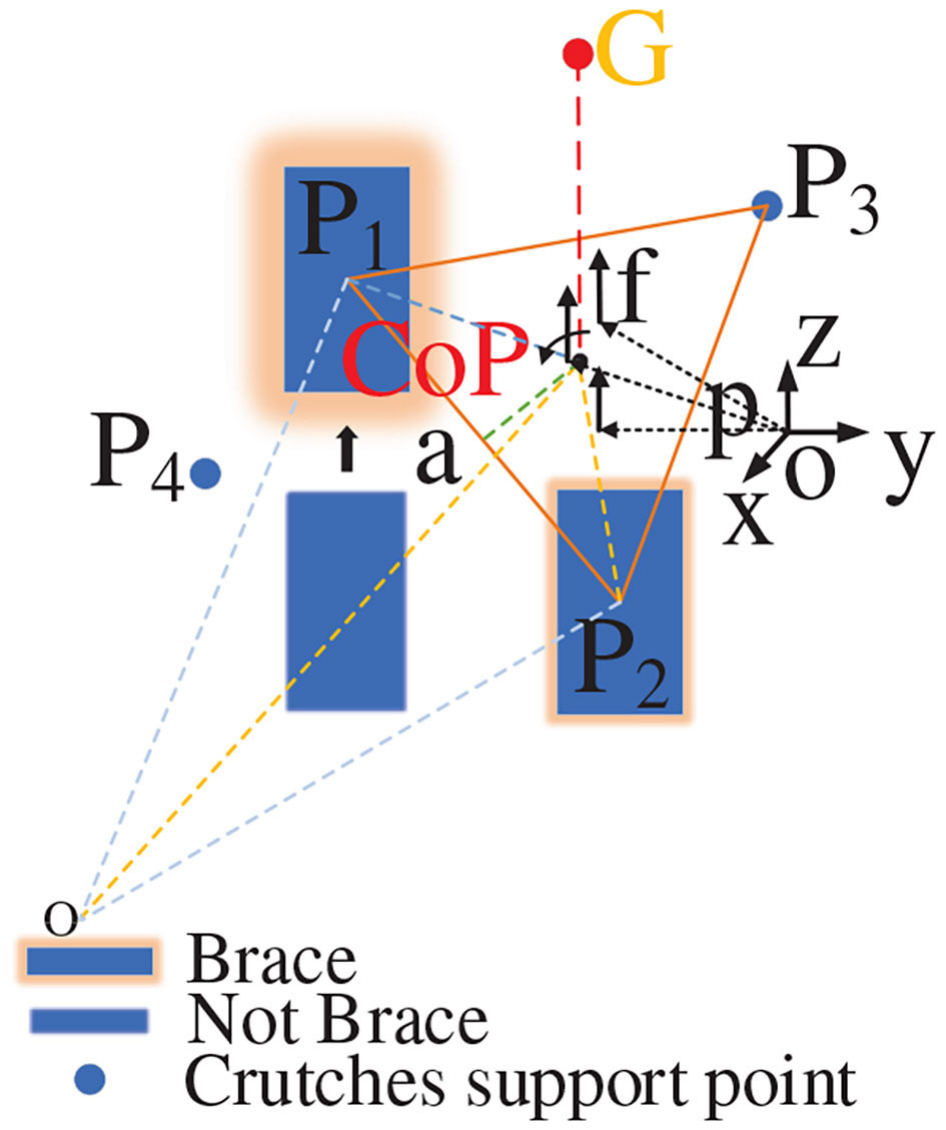
Zero moment point.

The entire movement process will only produce plantar pressure during the incomplete support stages. The main part of the full leg support stage is the forward force balance (Moraes et al., 2019). When the three supporting points form a triangle and the barycenter falls within the triangle, the stable state achieves at this time. The ZMP theory is used to calculate the overall pressure center point position of human–exoskeleton system during walking. The ground force is regarded as the discrete force set *f*_*i*_ ∈ *S*(*i* = 1, 2, ..., *N*) acting on *p*_*i*_, as indicated in Figure 5.

The moments around the point *p* are expressed as:

(9) $$\begin{array}{r} \tau =\overset{N}{\underset{i=1}{\sum }}\left(p_{i}-p\right)\times f_{i} \end{array}$$

Because the equivalent moment of force for the ZMP is zero, the foot does not rotate around the ground vertical axis (does not cause slipping). At this time, the ZMP position is:

(10) $$\begin{array}{r} p=\frac{\sum_{i=1}^{N}p_{i}f_{iz}}{\sum_{i=1}^{N}f_{iz}} \end{array}$$

In the formula, when *f*_*i*_*z* is the case in which *f*_*i*_ forms a triangular support in the vertical direction of the z axis, the ZMP position is:

(11) $$\begin{array}{r} p=\frac{p_{L}f_{L}+p_{R}f_{R}+p_{g}f_{g}}{f_{L}+f_{R}+f_{g}} \end{array}$$

In the above, *p*_*L*_, *p*_*R*_, and *p*_*g*_ represent the ZMP position obtained by the left foot, right foot, and crutch, respectively. By means of calculation, the center of pressure (CoP) and ZMP are almost coincident or coincident. We measure the shortest distance from the CoP to triangle the support side. We list one of these here as follows:

(12) $$\begin{array}{r} \alpha_{Ca}=d_{C⊥P_{1}P_{2}} \end{array}$$

(13) $$\begin{array}{r} d_{C⊥P_{1}P_{2}}=|\vec{OC}-\vec{OP_{2}}-\left(\vec{OP_{1}}-\vec{OP_{2}}\right)\cdot \left(\vec{OC}-\vec{OP_{2}}\right)\vec{e}| \end{array}$$

In order to ensure stability of human–exoskeleton system, we analyzed the stability of different triangular support structures.

**Figure 6:**
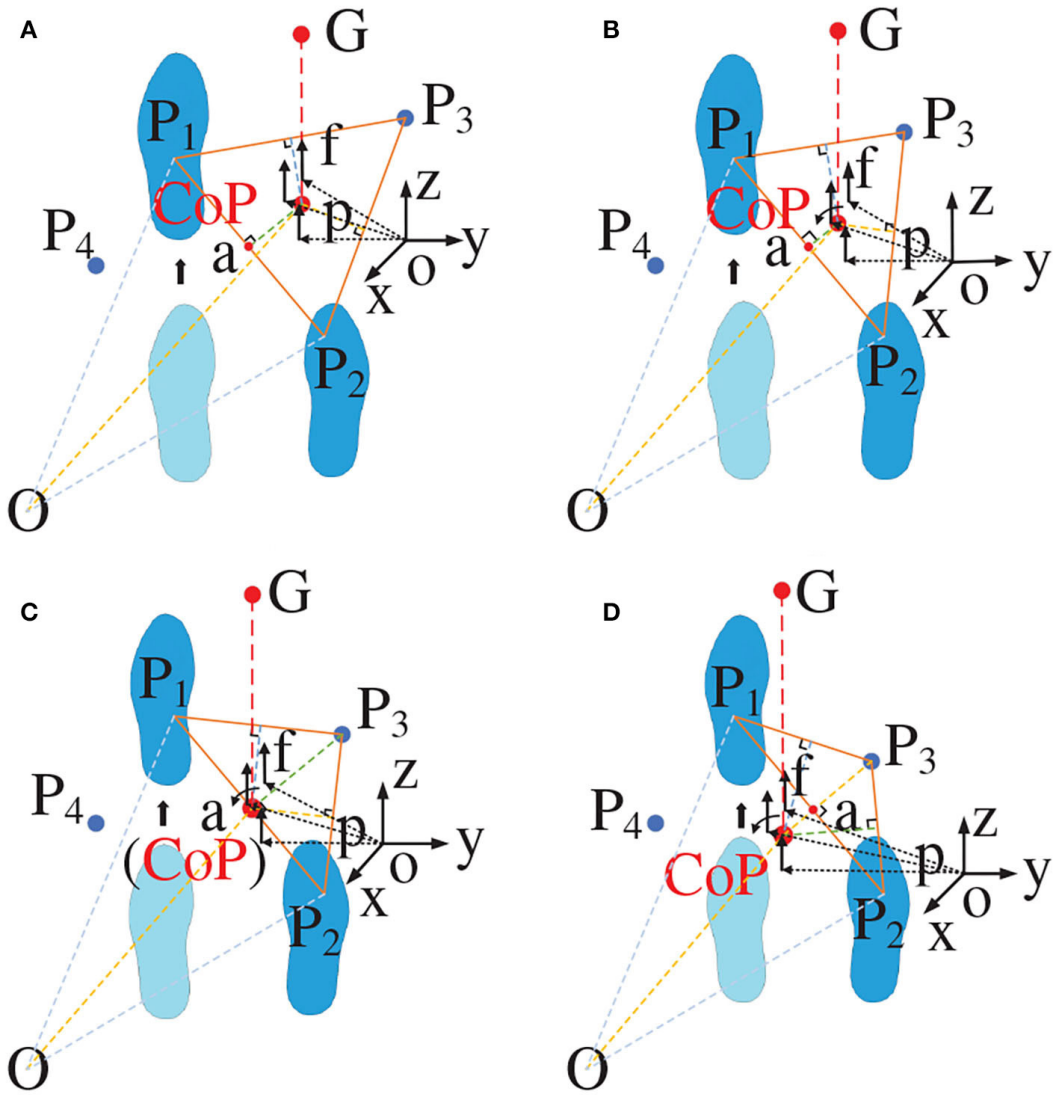
Triangular force analysis. **(A)** The barycenter falls in the center of the triangle; **(B)** The barycenter falls in the non-center of the triangle; **(C)** The barycenter falls in edge of the triangle; **(D)** The barycenter falls out of the triangle.

When the position of τ falls within the triangle formed by the support points, the value of α_*C*_*a*__ is >0, and stability is indicated. The barycenter will fall in the center of the triangle only when the vertical distance between each point of the CoP point and triangle is sufficiently small and all distances are equal, indicating that the three supporting points are evenly stressed, as shown in Figure 6A. When the three support points formed a normal acute triangle, as indicated in Figure 6B, the vertical distance between each point of the CoP point and the triangle is unequal, indicating that the force of the three support points is not uniform in this state. When the three support points form a right triangle, as indicated in Figure 6C, the force of the three support points is also uneven as the barycenter of the triangle falls on the oblique side, which constitutes a critical stable state. When the triangle formed by the three support points is an obtuse triangle, as indicated in Figure 6D, the barycenter of the triangle has fallen outside the triangle, constituting a tendency to dump. The stability threshold for walking at each step must be controlled within a safe range because an uneven force will accelerate muscle fatigue and soreness, resulting in a decrease in stability and affecting the rehabilitation effect.

## 2.2. sEMG Interface

A neural interface is constructed based on sEMG, which aims to accomplish the intention recognition and muscle fatigue estimation. Because of the lack of sEMG in lower limb for paraplegics, the sEMG signals and angle signals from the upper limbs are collected. The sEMG and angle signal of the subject were simultaneously collected while the subject wearing the SIAT exoskeleton was normally walking or triggering the gait conversion. One of the planned motions was listed, with the right elbow joint bent at not <90° as the triggering signal. This action is used as trigger because that paraplegics can perform this action fair easily in the standing posture. Moreover, considering that sEMG will be used for further intention recognition, this action will not conflict with other gait switching actions. The acquired joint angle was used as an auxiliary confirmation angle value to achieve more accurate intention recognition.

### 2.2.1. Motion Intention Recognition

To recognize the motion intention better, this research injected the LSTM deep learning model. A deep motion pattern identification model based on the LSTM structure is proposed to study the inherent characteristics of joint and sEMG from a time perspective (Miikkulainen et al., 2019). The LSTM model contains input, forget, and output gates (Zeiler, 2012), as well as one or more self-connected memory cells, which allows the cells to reset data when the network needs to forget useless inputs (Chang and Lin, 2011). The LSTM memory cells can store and access information over long periods.

The most important component in the LSTM neural network is the state unit $s_{i}^{\left(t\right)}$:

(14) $$\begin{array}{r} s_{i}^{\left(t\right)}=f_{i}^{\left(t\right)}s_{i}^{\left(t-1\right)}+g_{i}^{\left(t\right)}\sigma \left(b_{i}+\underset{j}{\sum }U_{i,j}x_{j}^{\left(t\right)}+\underset{j}{\sum }W_{i,j}h_{j}^{\left(t-1\right)}\right) \end{array}$$

The input of LSTM network is given as:

(15) $$\begin{array}{r} g_{i}^{\left(t\right)}=\sigma \left(b_{i}^{g}+\underset{j}{\sum }U_{i,j}^{g}x_{j}^{\left(t\right)}+\underset{j}{\sum }W_{i,j}^{g}h_{j}^{\left(t-1\right)}\right) \end{array}$$

The forgetting layer output is given as:

(16) $$\begin{array}{r} f_{i}^{\left(t\right)}=\sigma \left(b_{i}^{f}+\underset{j}{\sum }U_{i,j}^{f}x_{j}^{\left(t\right)}+\underset{j}{\sum }W_{i,j}^{f}h_{j}^{\left(t-1\right)}\right) \end{array}$$

The LSTM network cell output is given as:

(17) $$\begin{array}{r} h_{i}^{\left(t\right)}=\text{tanh }\left(s_{i}^{\left(t\right)}\right)q_{i}^{\left(t\right)} \end{array}$$

The LSTM network output is given as:

(18) $$\begin{array}{r} q_{i}^{\left(t\right)}=\sigma \left(b_{i}^{o}+\underset{j}{\sum }U_{i,j}^{o}x_{j}^{\left(t\right)}+\underset{j}{\sum }W_{i,j}^{o}h_{j}^{\left(t-1\right)}\right) \end{array}$$

where b, U and W are the offset weights of the LSTM cells, input weights, and forget gates, respectively. Moreover, *x*^(*t*)^is the current input, while *h*^(*t*)^ is the current hidden layer vector containing the output of all LSTM cells.

The current memory is written as:

(19) $$\begin{array}{r} C_{t}^{\prime }=\text{tanh }\left(W_{i,j}^{c}x_{j}^{c}+U_{i,j}^{c}h_{j}^{\left(t-1\right)}+b_{i}^{c}\right) \end{array}$$

The current value for the states of the memory cells is given as:

(20) $$\begin{array}{r} C_{t}=f_{i}^{\left(t\right)}C_{t-1}+g_{i}^{\left(t\right)}C_{t}^{\prime } \end{array}$$

The memory block output is given as:

(21) $$\begin{array}{r} h_{j}^{\left(t-1\right)}=q_{i}^{\left(t\right)}\text{tanh }\left(C_{t}\right) \end{array}$$

**Figure 7:**
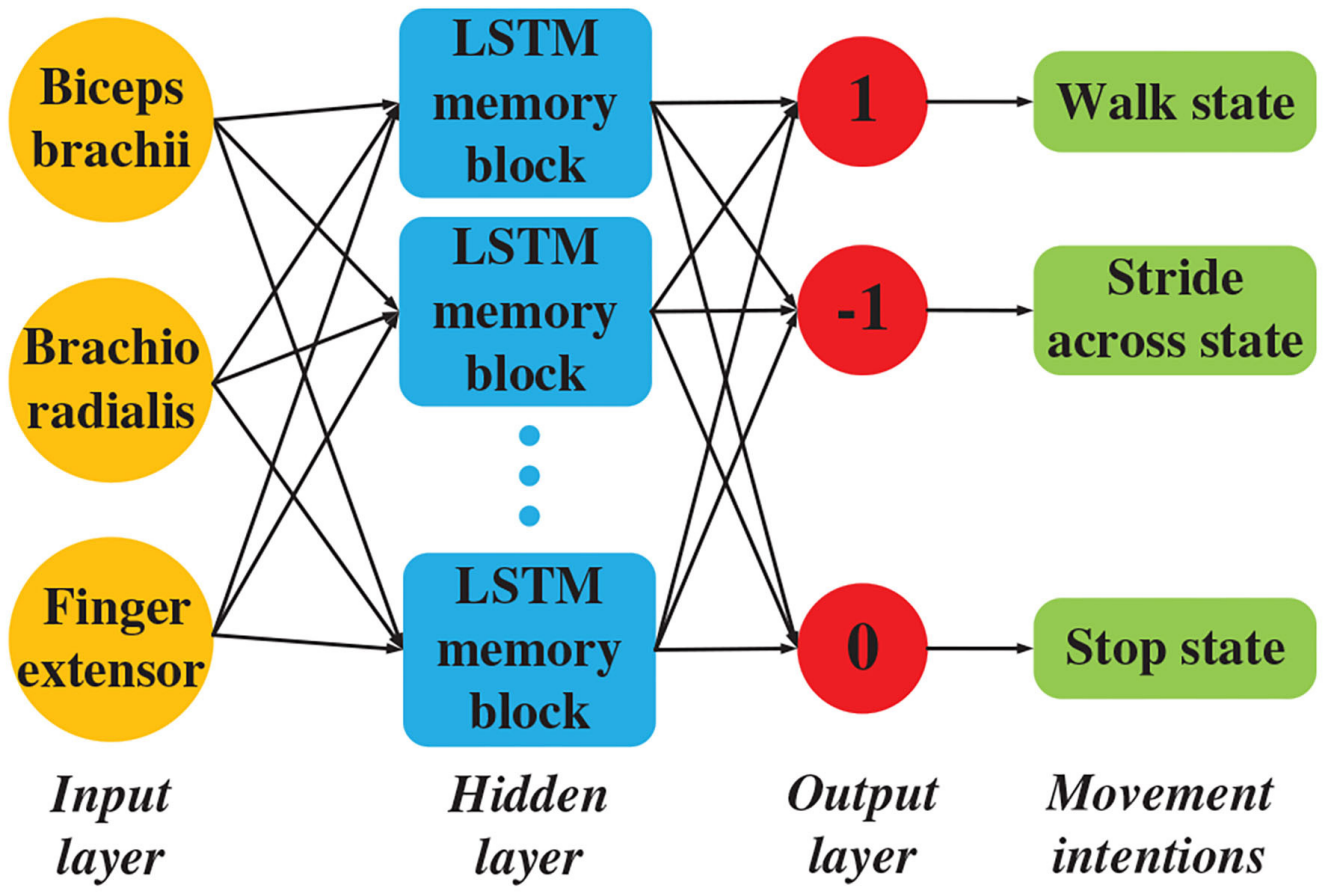
LSTM network block diagram.

Based on the LSTM neural network framework, the neural network for motion intention recognition is constructed. Multiple LSTM cells can be stacked for more expressive power, as presented in Figure 7. In different states, the elbow joint angle data and the sEMG signal of the selected muscles (the musculus biceps brachii, brachioradialis, and finger extensor) are collected. Based on the collected data, the movement intention is identified through the LSTM neural network model, which is built using TensorFlow^Ⓡ^. The neural network model is trained and verified by the obtained data. The exoskeleton system is controlled by the optimal model through predicting the motion intention of wearer. The LSTM neural network structure consists of one input layer, two hidden layer, and one output layer. The input layer is composed of three nodes corresponding three selected muscles. The number of neuron nodes in hidden layer is 128, and the hidden layer uses ReLU as the activation function. The dropout probability of nodes is set to 0.5. Taking advantage of dropout can make the LSTM network model more robust and avoid the problem of over-fitting (Zeiler, 2012). In order to prevent gradient drop, the adaptive moment estimation algorithm and weighted average method are utilized in this research. The LSTM model achieves optimization by using the following paraments: train errors = 0.004583, train costs = 2.511049, valid errors = 0.001667, and valid costs = 0.198940. We explain the different LSTM neural networks that are used for accuracy comparisons (Chu et al., 2007). The softmax layer is selected as the output layer. The output layer is composed of three nodes, which correspond with the three gait categories.

### 2.2.2. Muscle Fatigue Estimation

In this research, muscle fatigue is quantified based on sEMG through the neural interface and the wavelet packet energy entropy (WPEE) is utilized to quantify the muscle fatigue. WPEE not only has the advantages of wavelet transform but also tests the overall representation of the signal from the perspective of the system. The complexity of the frequency component signal and the dynamic characteristics of the signal are given. The wavelet packet decomposition and composition are completed with daubechies wavelet basis function (db1), which have good smoothness and compactness (Nakashima and Kushida, 2019).

After wavelet packet decomposition, the reconstructed signal energy of (*i, j*) is calculated as:

(22) $$\begin{array}{r} E_{i}^{j}=\overset{M}{\underset{k=1}{\sum }}|d_{i,j}^{k}{|}^{2} \end{array}$$

where $d_{i,j}^{k}$ denotes the coefficient of the *k*th decomposition node (*i, j*), and *M* presents the number of points in the decomposed signal sequence. Then, the relative value of energy in single frequency band, which presents the each band energy distribution of signals in overall frequency range, can be acquired by normalizing the energy:

(23) $$\begin{array}{r} P_{i}^{j}=\frac{E_{i}^{j}}{\sum_{j=0}^{2^{i}-1}E_{i}^{j}} \end{array}$$

where *j* ∈ [0, 2^*i*^ − 1]. The probability is also called relative wavelet packet energy. Combining the energy distribution of wavelet packet decomposition coefficient with information entropy, the WPEE is defined as:

(24) $$\begin{array}{r} \text{WPEE }=-\overset{2^{i}-1}{\underset{j=0}{\sum }}P_{i}^{j}\ln\;P_{i}^{j} \end{array}$$

WPEE can quantitatively measure the order and disorder of sEMG frequency distribution. If the sEMG energy is concentrated in one sub-band, the WPEE is 0, that is, the sEMG is orderly; on the contrary, if the sEMG energy is randomly dispersed in each sub-band, the sEMG is disorderly. Therefore, if muscles are more fatigue, the frequency of sEMG is more compressed to low frequency (Guan et al., 2017; Nakashima and Kushida, 2019), contributing to a decrease in WPEE. The values of WPEE are first normalized and then statistically stratified to support visualization and comparison of the muscle fatigue quantitative results.

## 3. System Setup

**Figure 8:**
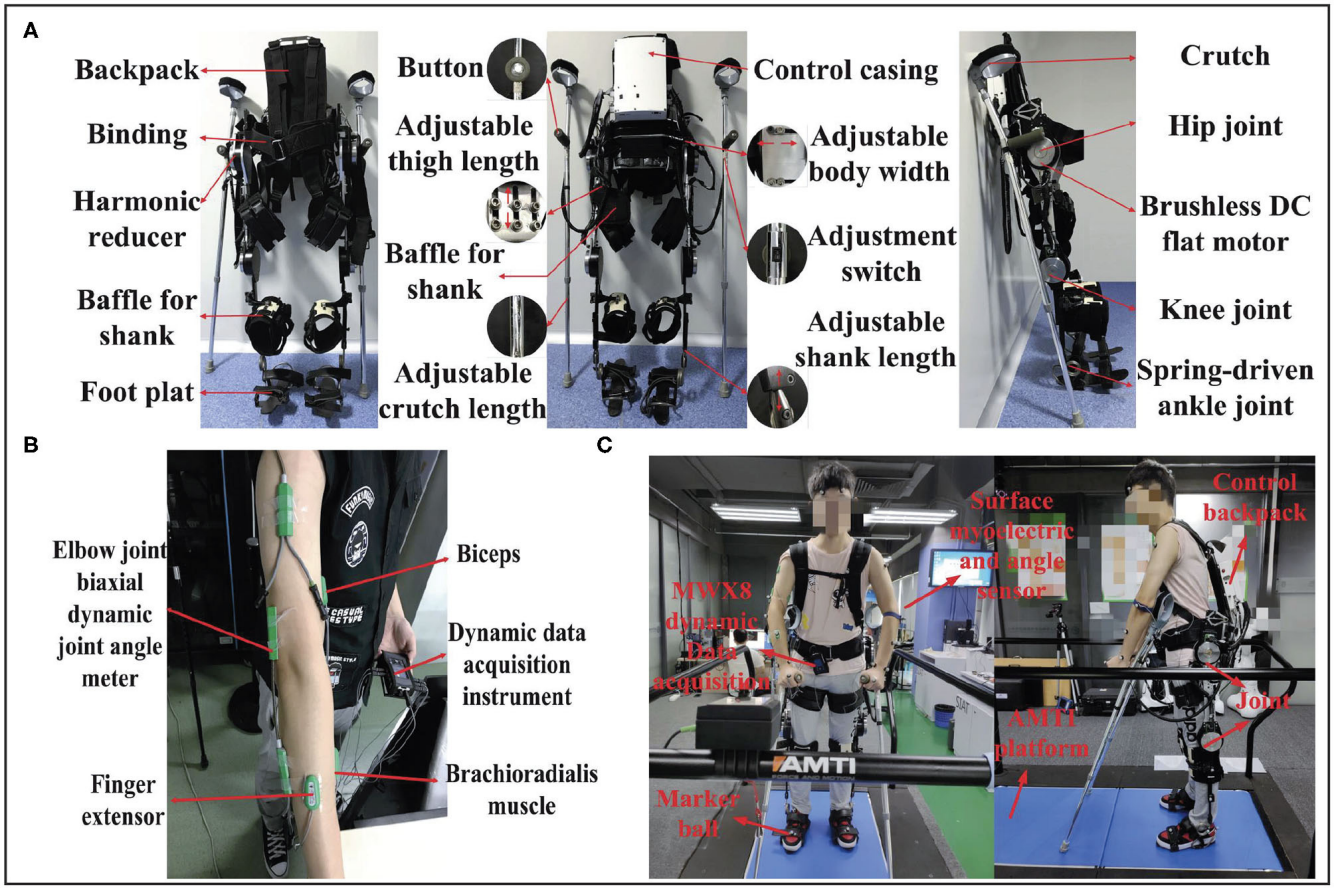
System setup: **(A)** SIAT Lower-limb Rehabilitation Exoskeleton Robot; **(B)** sEMG acquisition system and selected muscles; **(C)** VICON BONITA 10 environment, including Vicon camera, Vicon sole, and AMTI platform.

The main equipment in this research includes SIAT Exoskeleton, Biometrics sEMG acquisition system and Vicon Nexus motion capture system, as illustrated in Figure 8.

### 3.1. SIAT Exoskeleton

The SIAT exoskeleton robot, which is driven with two geared brushless motors for each leg, is designed for the disabled. This robot was developed by the Shenzhen Institutes of Advanced Technology, Chinese Academy of Sciences. The SIAT lower-limb rehabilitation exoskeleton robot is illustrated in Figure 8A. The SIAT exoskeleton robot includes hip, and knee joints, with a total of four DoFs. It can provide assistance to the hip and knee joints in the sagittal plane. Furthermore, the ankle joint is equipped with a spring mechanism to ensure a maximum contact area between the sole and ground to maintain stability. The encoders can obtain the angle of the hip and knee joints and crutches are used to ensure overall balance of the exoskeleton.

The SIAT exoskeleton consists of a mechanical frame, drive system, control system, and sensing system. The total weight is approximately 15 kg. The control unit is a Windows microcomputer that is placed in a white backpack on the back. The use of the microcomputer offers numerous advantages including the ability to optimize software specifically for the exoskeleton robot, while in use or copied to the microcomputer for direct use (Yan et al., 2018).

### 3.2. Biometrics

The portable sEMG signal acquisition system (Biometrics PS850) includes a signal acquisition device (DataLog) and management software that is specifically designed for sEMG measurements. The sEMG acquisition device includes eight independent programmable analog channels and two digital channels. The used sampling rate is set to 500 Hz in this research. Using the DataLog data acquisition memory, the collected signal records can be stored and analyzed. Three analogy channels are used to collect sEMG and a wired twin-axis goniometer is utilized to collect angle signal. The surface myoelectric collectors were attached to three selected muscles (musculus biceps brachii, brachioradialis, and finger extensor on the right arm), and the angle sensor was attached to the elbow joint. The sEMG acquisition system and selected muscles are illustrated in Figure 8B.

### 3.3. Vicon Nexus

The full set of equipment (Vicon BONITA 10) from Vicon Nexus is utilized for motion data acquisition and verification. The center of gravity movement trajectory and movement pressure of the exoskeleton robot during the walking process were detected by the pressure-measuring device on the running platform. The Vicon motion capture system captured the real-time motion of the exoskeleton robot wearer, detected the motion posture, and determined the wearer's center of gravity movement trajectory.

Vicon is a 3D optical motion capture system developed by Oxford Metrics Limited in the United Kingdom. VICON BONITA 10 environment includes Vicon cameras, a Vicon sole, and the AMTI platform, as shown in Figure 8C. Every part of the 3D optical motion capture system are connected via a network to provide real-time optical data, and it can be used for real-time online or off-line motion capture and analysis. The working principle is based on a reflective capture system, which requires reflective balls (markers) on the wearer. When the Vicon camera emits red light onto the reflective ball, the reflective ball will reflect red light of the same long wavelength to the camera. Therefore, the capture camera can determine the two-dimensional coordinates of each reflective ball. Following Vicon's control software processing, the 3D coordinates and the trajectory of each reflective ball can be obtained. In this study, we used six cameras to capture the motion pose.

## 4. Experimental Works and Results

Three healthy male subjects participated in the experiments of this work and voluntarily signed an informed consent form, which was approved by the Medical Ethics Committee of Shenzhen Institutes of Advanced Technology [(SIAT)-IRB-170315-H0142].

### 4.1. Experimental Design

When people with disabilities in the lower limb suddenly stand up with exoskeleton, it is difficult for them to master balance. Moreover, paraplegic patients do not trust the exoskeleton robot when they wear the exoskeleton for the first time. In this case, the support point position of the crutches is particularly important.

**Figure 9:**
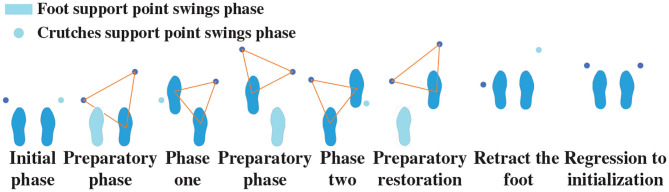
Supporting phase structure in walking condition.

During one movement cycle, the crutches are regarded as support points. Therefore, the human–exoskeleton system forms a quadruped state. During a gait cycle, the right crutch is first moved while lifting the left leg. With the left crutch as the support point, the right leg is lifted. Then, as the right crutch is moved to become the support point, the left leg is lifted. Finally, the crutches are retracted on the right-hand side to complete a periodic gait. It is necessary to form a three-point steady state during the movement and a four-point steady state in the stopping phase. Under normal circumstances, it is difficult to guarantee balance in the walking condition, and it is necessary to form a stable triangular support point. As shown in Figure 9, one complete period gait consists of a series of gait phases and states. Two-crutch points and supporting feet are required to form an equilateral triangle in the transition phase to maintain the stability of the human–exoskeleton system.

In experiments, the subject was attached to the exoskeleton through soft bandages. While the human exoskeleton was walking, the weight was transmitted to the supporting legs and crutches. Walking with different gaits was performed on the Vicon 3D force-measuring platform. In total, 10 datasets were collected for each gait group.

To control exoskeleton robot based on motion intention of the subject in real time, the sEMG of selected muscles are simultaneously collected. The gait of the exoskeleton robot is then switched according to the identified results derived from the optimized LSTM model. A real-time communication connection between the intention recognition and the exoskeleton robot is constructed. We use Visual Studio (VS) to establish the exoskeleton Windows presentation foundation (WPF) control interface, establish intention recognition based on the LSTM model on MATLAB^Ⓡ^, build a real-time communication connection between the sEMG signal acquisition system and MATLAB^Ⓡ^ to make it run simultaneously, and use the IP address and port to establish a real-time communication connection with the exoskeleton WPF control interface.

**Figure 10:**
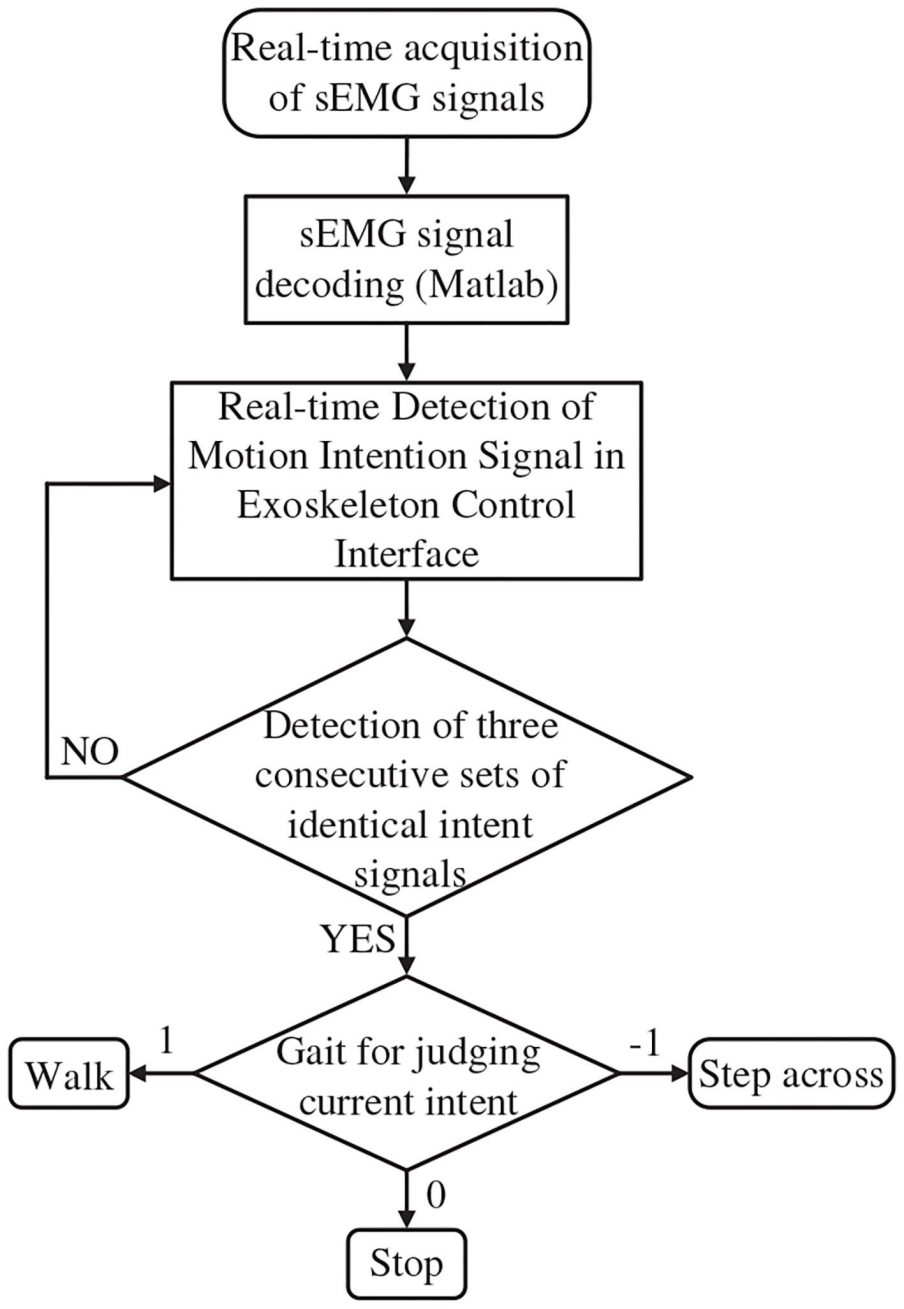
The flowchart of real-time intention recognition.

Real-time training is conducted through machine learning by collecting sEMG signals of stopping, walking, and stride across states. The recognized signal is then transmitted to the VS program through real-time communication interface. The real-time intention recognition flowchart is shown in Figure 10.

The pressure data were monitored in real time through the 3D force measuring treadmill and the real-time motion trajectory of the subject was determined through the Vicon dynamic capture system, as illustrated in Figure 3. Eight different gaits in this experiment, and planned and unplanned walking were performed in each gait. The equilateral triangle support structure is considered as the planned gait and the rest of the triangle support structure as the unplanned gait. The data were collected and compared to verify that it is more stable in the planned walking state.

In addition, muscle fatigue experiments are conducted to demonstrate the advantage of our proposed gait switch method further. Each subject wearing the exoskeleton robot walks for 12 min (2 sessions × 6 min) using planned and unplanned gait switching method, respectively. The subject would have enough rest between each walking test to reduce the fatigue impact from prior test.

### 4.2. Results and Analysis

**Figure 11:**
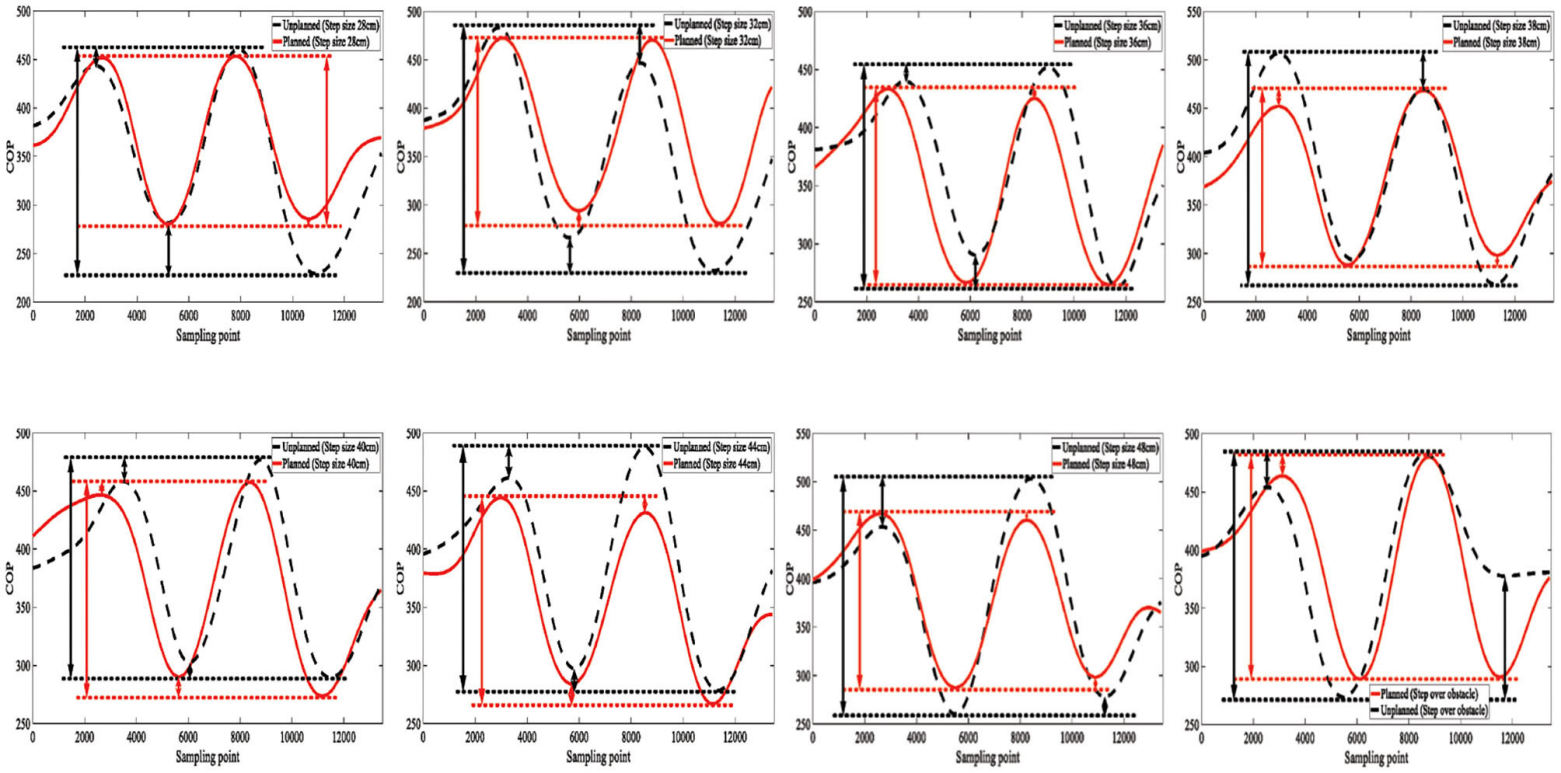
Comparation of CoP trajectory.

The CoP trajectories of different gaits based on the Vicon 3D force-measurement treadmill are compared. The comparation of each CoP trajectory is illustrated in Figure 11. It is found that stability is better while walking with the planned path. Under the planned gait switching method condition, the deviation between the similar wave peaks and the wave valleys is smaller in the different gait trajectories, the gait is smoother, and the center of gravity movement trajectory is smaller.

Through the reverse derivation of the human load bearing using the Vicon 3D force-measuring treadmill, the load bearing sizes of the ankle, knee, hip, and waist can be obtained. The gait with 28, 32, 36, 40, 44, and 48 cm steps size, and the gait of step over obstacle (stride cross) are utilized to be tested. Each subject walk with these gaits, and the average results deriving from each group of gaits are indicated in Table 2. By comparing the forces, we can observe that the planned gait results in greater conservation of strength. This is further verified by the muscle fatigue estimation results.

**Table 2:** Joints load bearing comparation between in planned and unplanned gait.

**Parameter**	**28 cm**	**32 cm**	**36 cm**	**40 cm**	**44 cm**	**48 cm**	**Stride cross**
Ankle joint force (N/kg) (Planned)	5.549	5.463	5.221	4.506	5.092	5.571	5.628
Knee joint force (N/kg) (Planned)	4.076	4.288	3.397	3.049	3.665	3.798	4.828
Hip joint force (N/kg)(Planned)	5.094	5.312	4.410	4.056	4.646	4.810	5.836
Waist force (N/kg)(Planned)	6.675	5.757	5.210	4.565	5.674	5.934	6.603
Wrist force (N/kg)(Planned)	4.192	4.256	4.680	4.265	4.141	4.396	4.376
Ankle joint force (N/kg) (Unplanned)	7.755	7.205	6.265	7.152	6.327	6.335	6.882
Knee joint force (N/kg) (Unplanned)	6.286	5.054	4.623	4.318	4.901	5.446	6.885
Hip joint force (N/kg)(Unplanned)	7.267	6.123	5.645	5.336	6.243	6.472	7.171
Waist force (N/kg)(Unplanned)	7.673	7.467	7.322	6.801	6.850	6.723	7.702
Wrist force (N/kg)(Unplanned)	5.343	5.301	6.052	6.138	5.445	5.354	5.536

After the sEMG is collected, the power frequency interference at 50 Hz had to be eliminated (Kim et al., 2019). Bandpass filtering of 10–500 Hz was also required after the interference was removed by infinite impulse response. We collected 214,000 sample points as training data for the collected samples, and each motion covered 40 datasets with a length of 1,000. The feature vectors were extracted from the sEMG data using the root mean square (RMS):

(25) $$\begin{array}{r} RMS=\sqrt{\frac{\sum_{i=1}^{N}|x\left(i\right){|}^{2}}{N}}=\sqrt{\frac{\left|x_{1}^{2}+x_{2}^{2}+\ldots +x_{N}^{2}\right|}{N}} \end{array}$$

The extracting feature causes the features between different signals to be more prominent and improve accuracy. The data could be mapped nonlinearly to a high-dimensional space to solve the linearity of the original space.

The objective function is expressed as:

(26) $$\begin{array}{r} \underset{\alpha_{i}⩾0}{\max}\underset{w,b}{\min}L\left(w,b,\alpha \right)=p^{*} \end{array}$$

where p^*^ represents the optimal value.

The corresponding classification function is given as:

(27) $$\begin{array}{r} f\left(x\right)=\overset{n}{\underset{i=1}{\sum }}\alpha_{i}y_{i}K\left(x_{i},x\right)+b \end{array}$$

where K(x,z) represents the construction kernel function. The extracted feature vector was affixed with the corresponding motion pattern tags and placed in the neural network as datasets for processing. Note that 80% of the data were used as the test set, and the remaining 20% were used as the verification set. The correct rate was verified via cross-validation of different folds. The common machine learning method, including Supporting Vector Machine (SVM), Multiple Layers Perception Neural Networks (MLPNN), K-Nearest Neighbor (KNN), and Linear Discriminant Analysis (LDA), are compared with the LSTM model. The comparison of intention recognition rate is indicated in Table 3.

**Table 3:** Intention recognition rate comparison.

**Method**	**2-Fold (%)**	**4-Fold (%)**	**6-Fold (%)**	**8-Fold (%)**	**10-Fold (%)**	**12-Fold (%)**	**14-Fold (%)**
LSTM	96.5	98.0	99.2	99.4	99.4	99.5	99.5
SVM	94.0	95.0	95.0	95.3	96	96.5	96.5
KNN	85.5	90.0	91.7	92.4	90.1	90.0	89.5
MLPNN	91.0	91.3	92.5	93	93.9	97.0	94
LDA	86.4	85.6	90.1	87.9	85.0	86.4	86.4

**Figure 12:**
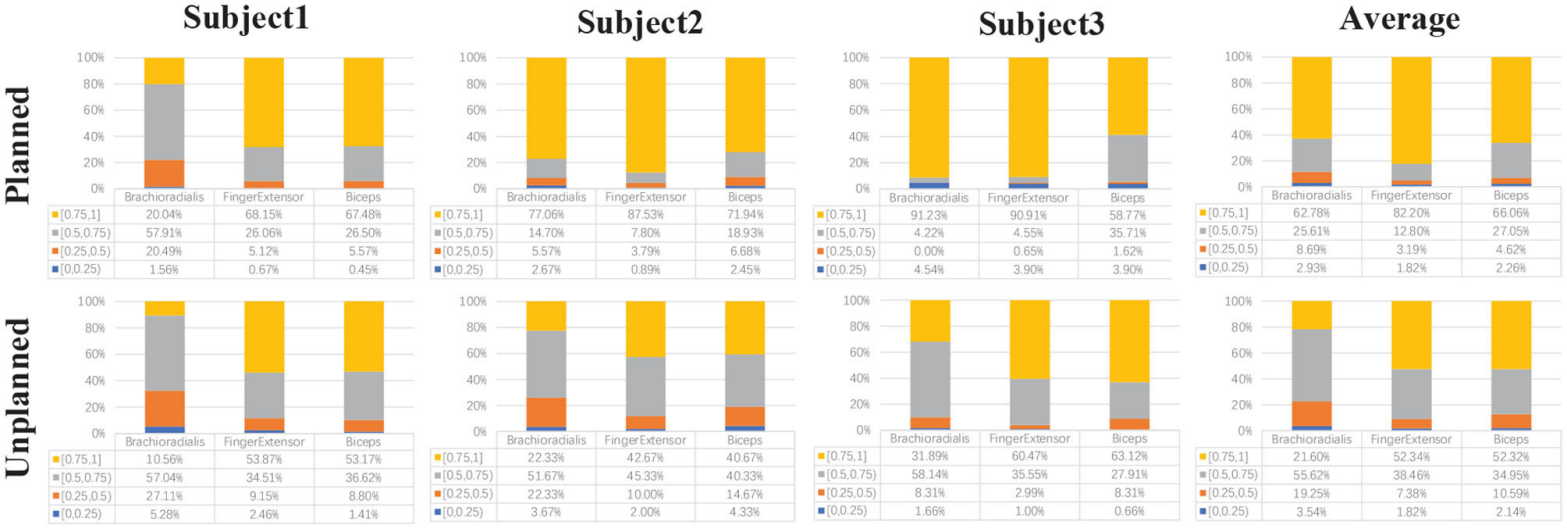
The comparation of muscle fatigue estimation based on wavelet packet energy entropy (WPEE) value.

During the muscle fatigue experiments, the gaits in different size are utilized to test. The subject wearing the exoskeleton robot walks back and forth in an open area about 10 m long and 3 m wide. The subject crosses an obstacle (21.5 × 13.5 × 10.5 cm) two times and turns around at the end of the path. The subjects are asked to move the body weight forward as much as possible to imitate the paraplegic. The muscle fatigue estimation results and analysis in gait with 36 cm are demonstrated as in Figure 12. The normalized WPEE value is divided into four layers and statistics are completed. The lower the WPEE value, the more severe the muscle fatigue. It is obvious that the proportion of high value WPEE in planned gait switching condition is more than that in unplanned gait switching condition. Therefore, we may safely claim that the muscle is more fatigue using the unplanned gait switch method.

## 5. Conclusions

In this research, an adjustable real-time stable gait switching strategy with a neural interface for the lower extremity exoskeleton robot is proposed. The gaits planning and stability analysis based on the human kinematics model for the SIAT lower limb exoskeleton are first demonstrated. A neural interface based on sEMG that realizes the intention recognition and muscle fatigue estimation is constructed. The stability of human–exoskeleton system and muscle fatigue of the wearers with the exoskeleton robot were tested through different gaits. The intention recognition accuracy based on the LSTM model was approximately 99%. The feasibility and efficiency of the proposed gait switching method is verified using the experimental results.

Although the proposed real-time gait switching strategy has shown advantages, several limitations of this study should be noted. First, the real-time gait switching method was only trained and tested using healthy subjects, and paraplegic patients may generate different locomotion features. Second, the most steady-state gait needs to be adjusted by online real-time gait feedback compensation. Moreover, the sEMG function of paraplegic patients may be incomplete and not easily obtainable. In future work, real-time gait feedback compensation adjustment should be considered to enable the exoskeleton robots to adapt to different walking environments.

## Data Availability Statement

The raw data supporting the conclusions of this article will be made available by the authors, without undue reservation.

## Ethics Statement

The studies involving human participants were reviewed and approved by the Medical Ethics Committee of Shenzhen Institutes of Advanced Technology. The patients/participants provided their written informed consent to participate in this study.

## Author Contributions

CW and ZG conceived the research project. CW and XW developed the SIAT exoskeleton. ZG, SD, BH, and YY designed and performed the experiments. ZG and SD preformed the data processing and analysis and wrote the paper. CW, XW, BH, and YY performed supports and discussions. All authors reviewed and approved the submitted paper.

## Conflict of Interest

The authors declare that the research was conducted in the absence of any commercial or financial relationships that could be construed as a potential conflict of interest.
